# Neural correlates of affective self-positivity bias in first and second languages

**DOI:** 10.3389/fpsyg.2026.1749078

**Published:** 2026-08-05

**Authors:** Chao Kong, Huanhuan Liu, John W. Schwieter, Francisco Muñoz

**Affiliations:** 1 Department of Psychology, Renmin University of China, Beijing, China; 2 Institute of Psychological and Brain Sciences, Liaoning Normal University, Dalian, China; 3 Key Laboratory of Brain and Cognitive Neuroscience, Dalian, Liaoning, China; 4 Language Acquisition, Cognition, and Multilingualism Laboratory/Bilingualism Matters, Wilfrid Laurier University, Waterloo, ON, Canada; 5 Department of Linguistics and Languages, McMaster University, Hamilton, ON, Canada; 6 School of Education, University College Dublin, Dublin, Ireland; 7 Center for Human Evolution and Behavior, Instituto de Salud Carlos III, Universidad Complutense de Madrid, Madrid, Spain

**Keywords:** bilingualism, emotion, fMRI, language context, self-positivity bias

## Abstract

**Introduction:**

An inherent positive bias emerges when individuals perceive themselves compared to when they perceive others. However, for bilinguals, it has been suggested that this self-positivity bias may be sensitive to the language in which information is presented.

**Methods:**

In this study, we examine this hypothesis among a group of Chinese-English bilinguals who performed a lexical decision task in their first (L1, Chinese) and second (L2, English) language. During the experiment, brain activity was recorded using functional Magnetic Resonance Imaging (fMRI).

**Results:**

Results showed faster responses and decreased activity in the right medial frontal gyrus (RMFG) for self-positivity compared to other-positivity conditions in the L2 context. However, this identified self-positivity bias in the L2 elicited less engagement of the RMFG than that in the L1.

**Discussion:**

These findings reveal distinct mechanisms across languages that underpin the self-positivity bias. Self-positivity bias is processed more embodied in an L1, drawing on richer emotional associations, whereas self-positivity bias is processed in a disembodied way in an L2, which requires integration of affective and lexical information.

## 1 Introduction

Self-related information carries an intrinsically positive bias (Northoff and Hayes, 2011). Humans tend to attribute positive attributes to themselves more readily than to others (Chen et al., 2014; Zhong et al., 2012), a phenomenon known as the self-positivity bias. Using an explicit self-referential judgment task, Watson et al. (2007) asked participants to decide whether positive and negative affective trait words were “like me” or “not like me” and found faster responses for self-congruent pairings, namely positive words judged as “like me” and negative words judged as “not like me,” than for the reverse pairings. This bias has also been examined using more implicit paradigms. Such as reading a self- or other-related sentence with a positive or negative meaning (Fields et al., 2019; Fields and Kuperberg, 2015). In the lexical decision task, participants judged whether positive, negative, and neutral words associated with the self or others were real words or nonwords (Liu et al., 2023). Together, these findings suggest that positive information linked to the self is processed more fluently than self-negative or other-positive information in faster responses and easier semantic integration (Fields and Kuperberg, 2015).

The self-positivity bias appears to be sensitive to the language of presentation (Ivaz et al., 2019). Evidence concerning self-positivity bias across bilingual language contexts remains limited and mixed. For instance, Zhang et al. (2023) employed an Implicit Association Test among Chinese-English bilinguals and found a self-positivity bias in the first language (L1), marked by smaller N200 and larger P3-like/LPC components, but not in the second language (L2). The authors interpreted the reduced N200 as indicating lower conflict-monitoring or control demands, because self-positive pairings were more consistent with an automatic self-positive association. The enhanced P3-like/LPC response further suggested greater late-stage attentional and evaluative processing of self-positive information. Together, these findings indicate that the L1 context supports an automatic association between the self and positive information, whereas this bias is attenuated in a foreign-language context. By contrast, Liu et al. (2023) required unbalanced Chinese (L1) – English (L2) bilinguals to perform a lexical decision on real words or nonwords with learned associations between identity labels (self, other) and geometric shapes (circle, triangle). They reported a self-positivity bias in the L2, but not in the L1 context, indexed by a reduced N400 to self-positive relative to self-negative and an enhanced LPC to self-positive relative to self-negative and self-neutral. The results showed self-positive relative to self-negative easier semantic-affective integration, deeper late-stage evaluative processing, and greater attentional allocation in the L2 context. They interpreted this pattern as suggesting that L2 requires greater cognitive resources, may allocate more attention to language-related emotional stimuli, and thus make the interaction between emotion and self-concept more salient. Van Hugten and Van Witteloostuijn (2018) found a stronger self-serving bias in L2 English than in L1 Dutch, with students showing a greater tendency to attribute high test scores to their own ability while distancing low scores from internal causes by attributing them to external factors, such as task difficulty or bad luck. While languages are emotionally charged communication systems acquired through social interactions (Fan et al., 2018), it remains unclear how emotional words interact with the self-positivity bias differently in L1 and L2 contexts. Previous research has used perceptual matching, lexical decision, the Implicit Association Test, electrophysiological, and attributional measures, and the mixed findings may partly reflect differences in task demands and bilingual experience.

The medial prefrontal cortex (mPFC) may provide a neural basis for the self-positivity bias because this bias requires the integration of self-relevance and emotional evaluation. For instance, Herbert et al. (2011) found that self-related emotional words elicited greater neural activation in the mPFC and anterior cingulate cortex (ACC), regardless of their valence. Fields et al. (2019) also reported that when participants read positive social vignettes, the mPFC was more active in self-relevance versus other-relevance contexts. The mPFC has also been implicated in emotional evaluation, with different subregions contributing to affective and evaluative aspects of processing (e.g., Northoff et al., 2006; Roy et al., 2012). Dorsal-caudal regions of the ACC/mPFC are implicated in the appraisal and expression of negative emotional states, whereas ventral-rostral regions are more closely associated with the regulation of emotional responses through interactions with limbic structures (Etkin et al., 2011). Moreover, both the ventromedial PFC (vmPFC) and dorsomedial PFC (dmPFC) have been associated with cognitive components of empathy, a cognitive system that involves cognitive understanding of the other’s perspective (Shamay-Tsoory, 2011). Thus, the mPFC may be involved in both processing self-relevant information and perceiving emotional content. Accordingly, mPFC activity in self-positivity bias may reflect the flexible integration of self-relevance and emotional evaluation processing rather than a neural response specific to a single cognitive function.

On this backdrop, the current study compares the positivity effect between self and other in L1 and L2 contexts by analyzing behavioral performance and neural activity. We speculate that self-positivity biases in the two languages may involve different cognitive processes, which may be due to differential degrees of connection between language and emotion. There are two explanations for the differences in emotional information processing between L1 and L2: the link between language and emotion, and the automation of language. The L1 is often learned in the home environment, where children develop deep emotional bonds with their caregivers. Contrarily, the L2 is often learned in more formal, neutral classroom environments. So compared to the L1, L2 is less likely to be closely associated with emotions (Harris et al., 2006). Pavlenko (2012) proposes that proficient bilinguals use their L2s to process emotional words only at the semantic level, rather than the emotional level. According to language automation theory, due to the different immersion of different languages, L1s have a greater degree of automatic processing than L2s (Degner et al., 2012).

Unlike previous studies that employ tasks involving shape-identity associations (e.g., a square represents “self” and a circle represents “other”) (Ivaz et al., 2019; Sui et al., 2013; Sui et al., 2015), we employ a lexical decision task that includes identity prime words (e.g., “I,” “he”) in both languages. Accordingly, participants were unable to infer the true experimental intent, thereby eliminating the subjects’ response tendency induced by task orientation. We formulated the following predictions. First, because lexical processing is generally more automatized in L1, responses were expected to be faster in L1 than in L2. Second, because positive emotions form part of self-concept (Chavez et al., 2017; Herbert et al., 2011), self-related primes were expected to facilitate access to positive trait words relative to other-related primes, producing faster responses and more mPFC activation in the self-positive condition. We also predicted a Language × Identity interaction, indicating that the magnitude of this self-positivity bias would differ between L1 and L2. It is still not clear whether this interaction is similar in the L1 and L2 contexts.

## 2 Materials and methods

### 2.1 Participants

Thirty Chinese-English bilinguals, aged 18 to 25 years old (*M* = 21.1, *SD* = 1.8 years), from a Chinese University participated in the present study. For all the participants, Chinese was their first language (L1), and English was their second language (L2). They were right-handed, had normal or corrected-to-normal vision, and reported no history of neurological or psychiatric conditions or use of psychotropic medication. Data from one participant were discarded due to low image quality, and thus, the final sample consisted of 29 participants (20 females, 9 males). All procedures adhered to the ethical guidelines established by the China Association for Psychology.

Characteristics regarding the participants’ age of L2 acquisition (AoA), self-ratings of listening, speaking, reading, and writing abilities in both languages, and their score on a modified version of the Oxford Placement Test (OPT, Allan, 2004) are displayed in Table 1. Due to time restrictions, we randomly extracted 25 multiple-choice questions from the OPT and a cloze test with 25 questions. Of a total of 50 points, the participants’ mean score was 35.38 (*SD* = 5.68), placing them at an intermediate L2 proficiency level, as in previous studies on Chinese-English unbalanced bilinguals (Liu et al., 2016; Liu et al., 2020; Liu et al., 2022). Moreover, we asked the participants to rate their L1 and L2 language skills in listening, speaking, reading, and writing on a 6-point scale in which “6” indicated “perfect knowledge” and “1” indicated “no knowledge.” As shown in Table 1, paired-samples *t*-tests showed that participants rated their L1 stronger than their L2 across all four skills.

**Table 1:** Participants’ characteristics (Mean ± SD).

	**L1**	**L2**	***t***	***p***
Listening	5.52 ± 0.51	3.21 ± 0.62	17.467	<0.001
Speaking	5.07 ± 0.26	3.00 ± 0.76	14.803	<0.001
Reading	4.41 ± 0.95	2.72 ± 0.88	9.405	<0.001
Writing	4.72 ± 0.80	2.66 ± 1.40	8.706	<0.001
OPT	–	35.38 ± 5.68		
Age of Acquisition	<1	8.69 ± 2.58		

Language abilities in L1 and L2 were self-rated on a 6-point scale (1 = no knowledge, 6 = perfect knowledge). OPT = Oxford Placement Test.

### 2.2 Materials

We first identified 130 Chinese words describing human features (e.g., friendly, sad, young, excited, etc.) and their English translations. We then assessed them in terms of their emotional valence (negative, positive) and familiarity among a separate group of 24 Chinese-English bilinguals (19 female, age: 20–26 years, *M* = 23, *SD* = 1.74) with a similar L2 proficiency. To do so, we asked these individuals to rate the valence of Chinese words and English translations on a scale from 1 to 9 in which “1” indicated “extremely negative” and “9” indicated “extremely positive,” and to rate their familiarity with the words (“1” indicated “extremely unfamiliar” and “9” indicated “extremely familiar”). Based on these ratings, we selected 18 Chinese words (9 positive, 9 negative) and their English translations to form part of the experiment. Positive words were selected from those rated higher than 5, and negative words were selected from those rated lower than 5 (see Ye and Gawronski, 2016).

One-sample *t*-tests showed that the negative and positive valences of the selected words in both languages were significantly different than the mid-score of 5 (Chinese positive words: *M* ± *SD*: 6.65 ± 0.60, *t* = 8.286, *p* < 0.001; English positive words: *M* ± *SD*: 6.70 ± 0.56, *t* = 9.012, *p* < 0.001; Chinese negative words: *M* ± *SD*: 3.60 ± 0.51, *t* = −8.319, *p* < 0.001; English negative words: 4.01 ± 0.46, *t* = −6.395, *p* < 0.001). We then compared the valence and familiarity ratings between the two languages. As shown in Table 2, an independent samples *t*-test found that in both languages, the valence of positive words was significantly higher than negative words (L1: *t* = 11.702, *p* < 0.001; L2: *t* = 11.023, *p* < 0.001) and the difference in familiarity between positive and negative words was not significant (L1: *t* = 1.663, *p* = 0.116; L2: *t* = 1.991, *p* = 0.06). Finally, a paired-sample *t*-test revealed that there were no significant differences between overall mean valence ratings of L1 (*M* ± *SD*: 5.12 ± 1.67) vs. L2 experimental words (*M* ± *SD*: 5.35 ± 1.46), *t* = −1.841, *p* = 0.08; or between familiarity ratings of L1 (*M* ± *SD*: 7.39 ± 0.35) vs. L2 words (*M* ± *SD*: 7.21 ± 0.58), *t* = 1.164, *p* = 0.26.

**Table 2:** Comparisons of valence and familiarity ratings of experimental words (Mean ± SD).

	**L1 Positive**	**L1 Negative**	**L2 Positive**	**L2 Negative**
Valence	6.65 ± 0.60	3.60 ± 0.51	6.70 ± 0.56	4.01 ± 0.46
Familiarity	7.52 ± 0.28	7.26 ± 0.39	7.46 ± 0.30	6.95 ± 0.70

In addition to the 36 experimental words, we created 36 nonwords (18 in each language) to serve as fillers in the task. Chinese nonwords were formed by changing one component of a character from real words (e.g., changing the “子” into “亍” in the word “仔细,” which means “careful”). English nonwords were created by replacing one letter in a real word (e.g., “caroful”). Supplementary Tables S1, S2 provide a list of all experimental words and nonwords.

### 2.3 Procedure

The experiment was designed using E-Prime 2.0. The experimental session lasted approximately 28.5 min and included the 17-min experimental task, a 4.5-min structural image scan, and a 7-min resting state scan. The task was divided into two blocks, one for each language. The order of L1 or L2 blocks was counterbalanced across participants. Both blocks included a 10-s blank screen after the scanning started and lasted 8.5 min.

Prior to the experimental block in each language, a practice block in that same language was administered to the participants. The practice blocks consisted of 32 trials (24 real words, 8 nonwords) that did not appear in the actual experiment. Participants then proceeded to the experimental blocks. In each block, there were 2 warm-up trials and 90 experimental trials (72 real words, 18 nonwords). The 72 real-word traits included 18 self-positivity, 18 other-positivity, 18 self-negativity, and 18 other-negativity trials. The order of trait words was randomly presented, and each word appeared twice in the block. Previous studies have shown that self-positivity bias does not necessarily lead to other-negativity bias (see Fields et al., 2019; Xia et al., 2021; Zhang et al., 2023). Accordingly, the primary analyses in the present study focused on positive trials. Analyses of negative trials were also conducted and are reported in the Supplementary materials.

The experimental paradigm was adapted from self-referential tasks, in which affective personality trait words are preceded by pronoun cues indicating whether the subsequent information is self-relevant or other-relevant (e.g., Zhou et al., 2017). A lexical decision task was used as an indirect measure of the cognitive accessibility of the effects of the self- and other-related primes on the positive personality features. Previous research has shown that presenting self-related cues can activate individuals’ self-schema, which is generally positively biased, thereby facilitating the processing of congruent positive features (Fields and Kuperberg, 2015). Within a lexical decision task framework, it is expected that self-related priming increases the positive-trait word accessibility compared to other-related priming, which is manifested in faster response times.

**Figure 1:**
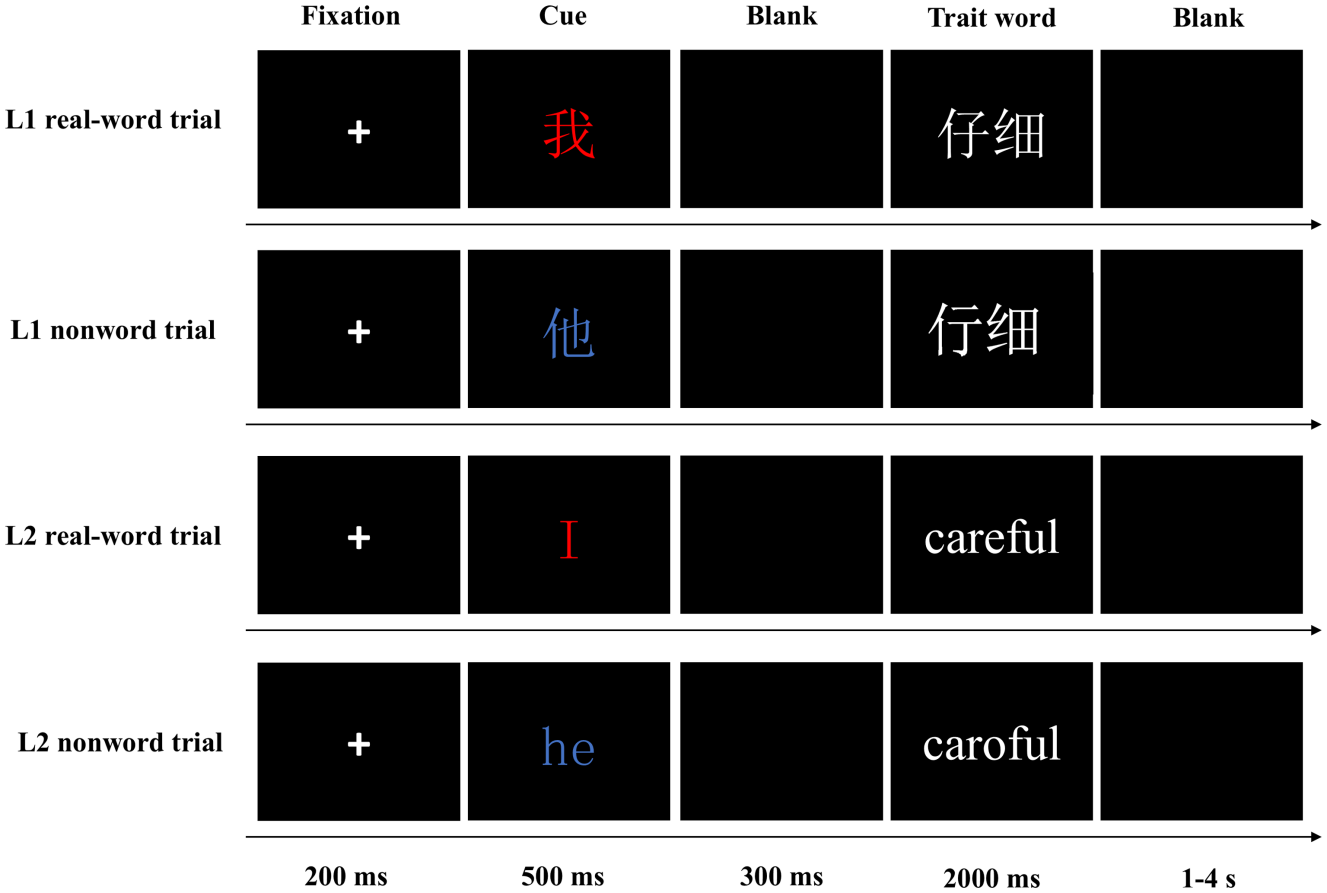
Experimental procedure of a single trial. Each trial consisted of a fixation cross (200 ms), followed by a cue indicating that subsequent information was self-relevant or other-relevant (self: “I/我”; other: “he/他,” 500 ms), a blank screen (300 ms), a trait word (2000 ms), and then a blank screen lasting between 1 and 4 s was presented before the next trial began. Participants were required to perform a lexical decision task, in which they indicated whether the trait word was a real word or a nonword. Chinese nonwords were formed by changing one component of a character from real words (e.g., changing the “子” into “亍” in the word “仔细,” which means careful). English nonwords were created by replacing one letter in a real word (e.g., “caroful”). 20% of the trials were nonword trials.

As shown in Figure 1, each trial began with a white fixation cross presented at the center of a black screen for 200 ms. This was followed by a prime cue referring to either the self (“我”, “I”) or another person (“他”, “he”) for 500 ms. For half of the participants, self-related primes were presented in red and other-related primes in blue, whereas the color assignment was reversed for the remaining participants. After a blank screen of 300 ms, a trait word stimulus (a real word or a nonword) appeared for 2000 ms. Participants were instructed to judge whether the presented stimulus was a real word by pressing “1” for real words and “2” for nonwords. Response key assignments were counterbalanced across participants. Finally, a blank screen lasting between 1 and 4 s was presented before the next trial began.

### 2.4 Behavioral data analyses

Given that accuracy was at ceiling (> 98%), we did not further analyze error rates. For response times (RTs), we conducted a two-way repeated-measures ANOVA with Language (L1, L2) × Identity (Self, Other) as factors. Incorrect responses and RTs that were ± 3 SD from the overall mean were excluded from the data analyses.

### 2.5 fMRI data acquisition

fMRI data were collected with a GE Discovery MR750 3 T scanner. Participants were instructed to minimize head movements during scanning. Functional images were collected using a T2*-weighted EPI sequence. Volumes covered the whole brain (repetition time = 2000 ms, echo time = 30 ms, flip angle = 90°, sequential acquisition = 33 axial slices, slice thickness = 3.5 mm, image matrix = 64 × 64, field of view = 256 × 256 mm^2^, voxel size = 4 × 4 × 3.5 mm^3^). Structural images were collected using a T1-weithted 3-D MPRAGE sequence (repetition time = 6.652 ms, echo time = 2.928 ms, flip angle = 12°, sequential acquisition = 192 slices, slice thickness = 1 mm, spacing between slices = 1 mm, image matrix = 256 × 256, field of view = 256 × 256 mm, voxel size = 1 × 1 × 1 mm^3^), to co-register them with the functional images (Liu et al., 2020).

### 2.6 fMRI data preprocessing

All fMRI data were preprocessed in DPABI software (Yan et al., 2016), which was based on Matlab R2013b. The first five functional images were discarded to avoid T1 saturation effects. For each participant, the origins of functional and anatomical images were aligned to the anterior commissure. The preprocessing procedure includes slice timing, 3D motion correction, segment, normalize, and smoothing. The anatomical images were segmented into gray matter, white matter, and cerebrospinal fluid to ensure that functional images best realigned to anatomical images. These images were then normalized to MNI space (Montreal Neurological Institute, resampled voxel-size 3 mm × 3 mm × 3 mm). Data were smoothed spatially with an isotropic Gaussian Filter of 6 mm × 6 mm × 6 mm full width at half maximum (FWHM). All in-scanner head motions of functional data were less than 3 mm or 3°.

### 2.7 fMRI data analyses

The SPM12 (Statistical Parametric Mapping software, Wellcome Dept. of Imaging Neuroscience, London, UK) software based on Matlab R2013b was used to run the first level analysis. The data was analyzed by a general linear model (GLM) with the following regressors: two for the Self-positivity and Other-positivity conditions in each language, one for nonwords, one for practice trials and trials with incorrect responses, four for other unconcerned conditions (Other-negativity and Self-negativity in each language), and six for in-scanner head motion parameters in each session. The time points refer to the onset time of the words. All regressors were convolved with the canonical hemodynamic response function. Contrasts were defined to compare the differences in brain activity between Language (L1 vs. L2) and Identity (Self vs. Other) in positive words, and activity involved in the four conditions (L1 Self-positivity, L1 Other-positivity, L2 Self-positivity, L2 Other-positivity).

In a second-level analysis, we tested for main effects of Language and Identity using a one-sample *t*-test. The design matrix consisted of one regressor for each participant. Given that the present study uses a within-subjects design and has different runs assigned to different languages, we used a factorial design (Sui et al., 2013) including four conditions to analyze the interaction between the factors Language and Identity. Statistical maps were thresholded using Gaussian Random Field (GRF) theory. GRF correction belongs to the family-wise error rate corrections approach (Woo et al., 2014) and allows for a strict correction using a single-voxel threshold of *p* < 0.005 and a cluster-level threshold of *p* < 0.05.

To determine the self- and other-biases in the L1 and L2, we conducted *t*-tests on the activation for both languages. To control for false-positives regarding interaction effects in the regions in the interaction between Language and Identity (see Table 3), a *p_uncorrected_* < 0.005 using small volume correction (SVC) in SPM12 was conducted (Seidel et al., 2018).

**Table 3:** Further results for the interaction between Language × Identity in positive words.

**RMFG condition**	**Cluster size**	**Brodmann areas**	***t***	**MNI coordinate**		
				**X**	**Y**	**Z**
L2: Other- vs. Self-positivity	94	46	5.42	30	39	24
Self-positivity: L1 vs. L2	13	46	3.76	39	39	12

RMFG = Right Medial Frontal Gyrus.

## 3 Results

### 3.1 Behavioral results

The two-way repeated-measures ANOVA (Language (L1, L2) × Identity (Self, Other) in positive words) revealed a significant main effect of Language, *F*(1,28) = 4.84, *p* = 0.032, *η_p_^2^* = 0.147, such that RTs were significantly faster in the L1 (*M* ± *SD*: 722 ± 143 ms) compared to the L2 (*M* ± *SD*: 757 ± 129 ms). The main effect of Identity was also significant, *F*(1,28) = 6.83, *p* = 0.014, *η_p_^2^* = 0.196, with faster RTs in the Self condition (*M* ± *SD*: 730 ± 129 ms) relative to the Other condition (*M* ± *SD*: 748 ± 130 ms).

**Figure 2:**
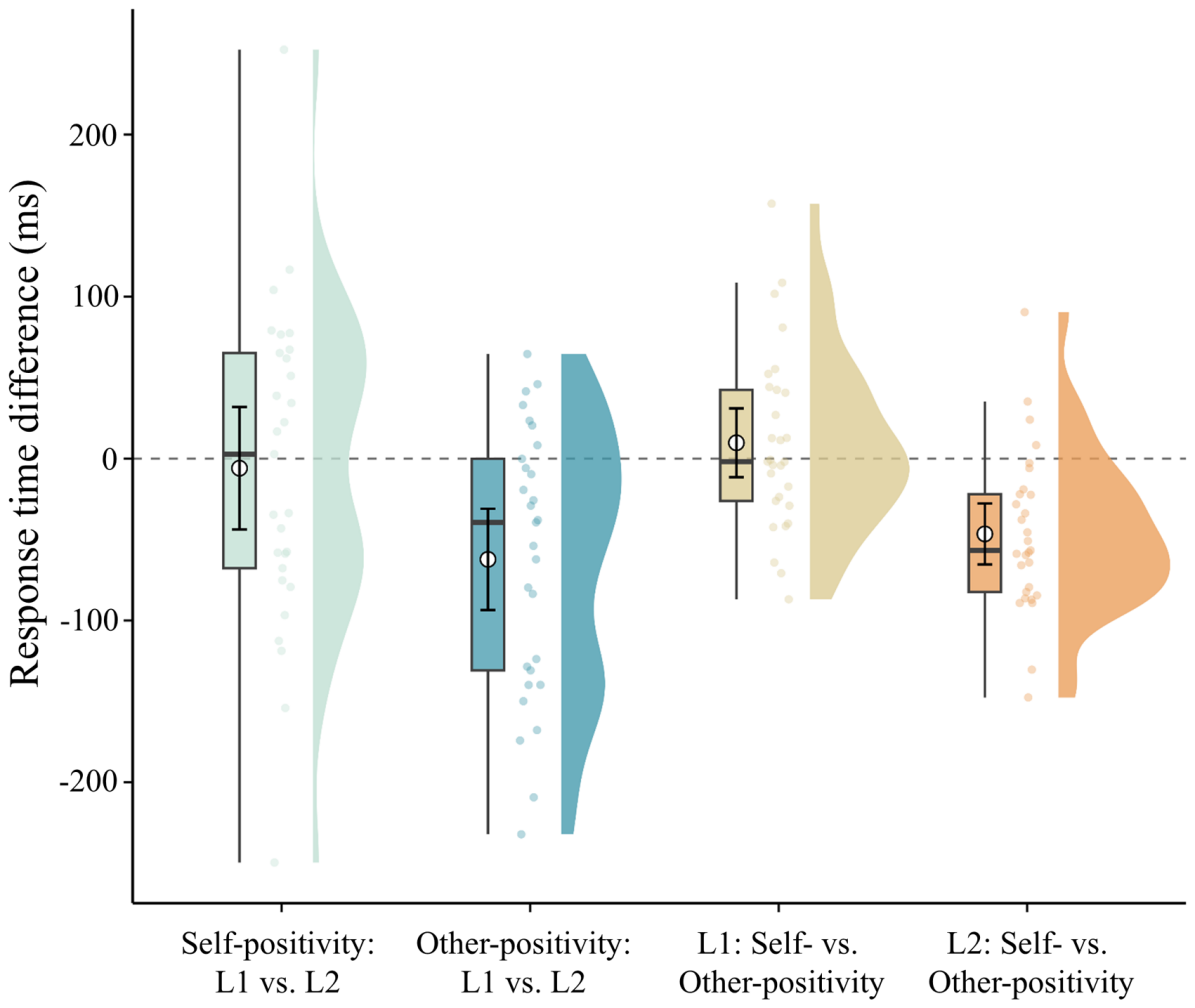
Within-subject response time differences across the positive condition. Half violins show the distribution of individual difference scores; boxplots summarize the median and interquartile range; white points indicate the mean difference; and error bars indicate 95% confidence intervals.

Importantly, the two-way interaction was significant, *F*(1,28) = 17.01, *p* < 0.001, *η_p_^2^* = 0.378. As shown in Figure 2, in the L1, the RT difference between Self-positivity and Other-positivity conditions was not significant [L1 Self (*M* ± *SD*): 727 ± 148 ms, L1 Other (*M* ± *SD*): 718 ± 144 ms, *F*(1,28) = 0.88, *p* = 0.356, *η_p_^2^* = 0.030], while in the L2, faster RTs were observed in the Self-positivity condition (*M* ± *SD*: 733 ± 129 ms) relative to the Other-positivity condition [*M* ± *SD*: 780 ± 133 ms, *F*(1,28) = 25.62, *p* < 0.001, *η_p_^2^* = 0.478]. The difference between L1 Self-positivity and L2 Self-positivity was not significant [L1 Self (*M* ± *SD*): 727 ± 147 ms, L2 Self (*M* ± *SD*): 733 ± 129 ms, *F*(1,28) = 0.104, *p* = 0.749, *η_p_^2^* = 0.004], while faster RTs were found in the L1 Other-positivity condition (*M* ± SD: 718 ± 144 ms) relative to the L2 Other-positivity condition (*M* ± SD: 780 ± 133 ms) was significant, *F*(1,28) = 16.62, *p* < 0.001, *η_p_^2^* = 0.372.

### 3.2 fMRI results

**Figure 3:**
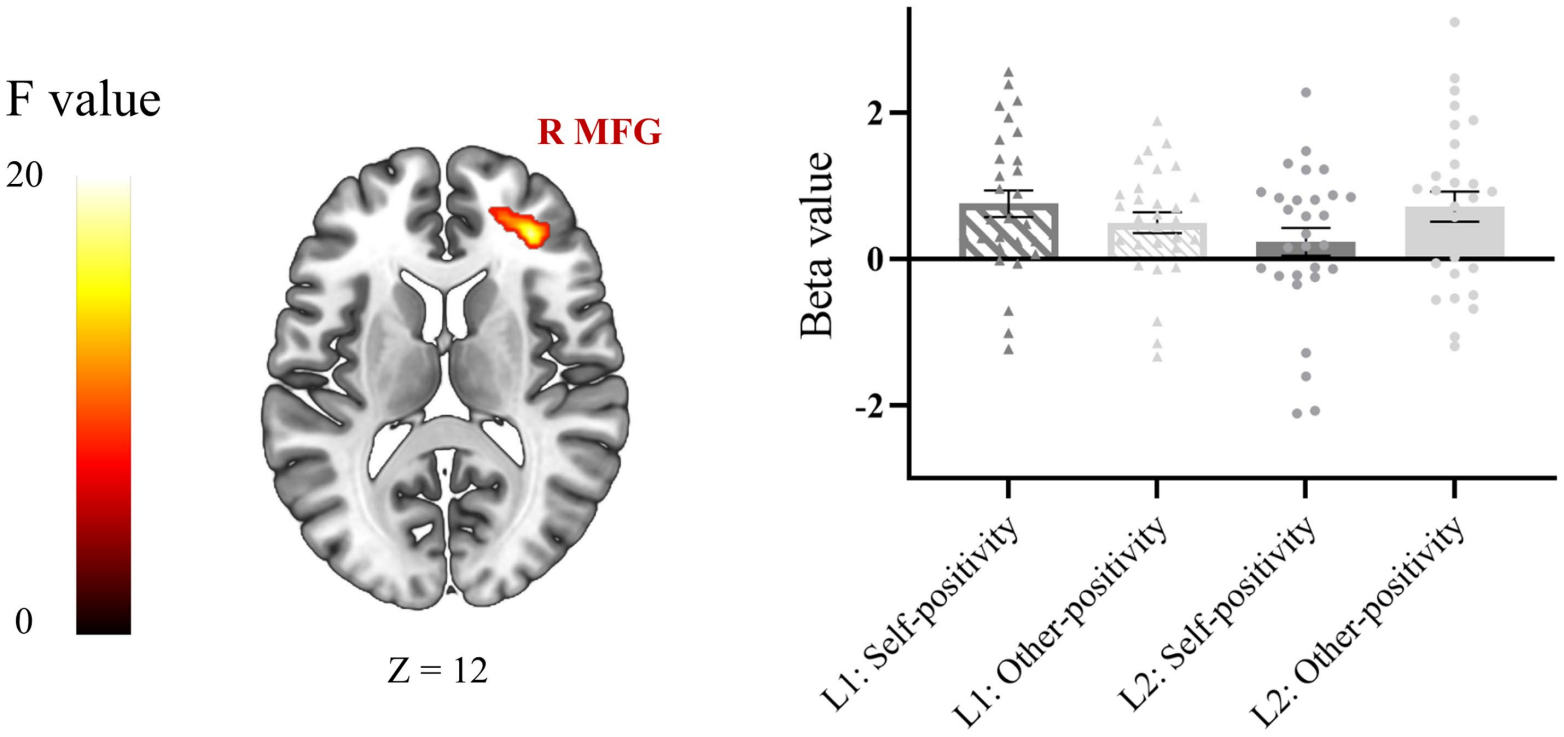
Language × Identity interaction localized in the RMFG, and corresponding beta values in each condition. RMFG = Right Medial Frontal Gyrus. Error bars indicate standard errors of means.

The factorial analysis revealed a significant two-way interaction between Language (L1, L2) × Identity (Self, Other) in the right medial frontal gyrus (see Table 4 and Figure 3). To explore this interaction, we split the analyses by Language and Identity respectively, and conducted *t*-tests between Self and Other (split by Language) and between L1 and L2 (split by Identity).

**Table 4:** The interaction of Language × Identity in positive words.

**Brain region**	**Cluster size**	**Brodmann areas**	***F***	**MNI coordinate**		
				**X**	**Y**	**Z**
RMFG	100	46	19.51	33	39	12

RMFG = Right Medial Frontal Gyrus.

In the L2, the activation difference between Other and Self was found in the right Medial Frontal Gyrus, showing greater activation in the Other-positivity condition compared to the Self-positivity condition in the L2, but not in the L1 (see Table 3). On the other hand, in the Self-positivity condition, the activation difference between the L1 and L2 was found in the RMFG, such that activation in the L1 Self-positivity was greater than L2 Self-positivity. There were no significant differences between the L1 and L2 Other-positivity conditions.

## 4 Discussion

The current study examined how language context modulates the self-positivity bias at both behavioral and neural levels among a group of Chinese-English bilinguals. The results revealed a dissociation pattern between L1 and L2, suggesting that distinct mechanisms across languages underpin the self-positivity bias.

In the L2 context, a behavioral self-positivity bias was observed. The other-positivity condition elicited slower responses than the self-positivity condition. Moreover, fMRI results revealed increased RMFG activation in the other-positivity condition compared to the self-positivity condition. In contrast, no significant behavioral or neural self-positivity bias was observed in the L1. However, the RMFG activation was higher in the L1 self-positivity condition than in the L2 self-positivity condition. Together, these results indicate that a self-positivity bias occurred in the L2 by recruiting the RMFG, suggesting that self-related processing in an L2 relies more on controlled empathy mechanisms compared to the more automatic processing observed in L1. This dissociation aligns with the Language (Dis)Embodiment theory (Caldwell-Harris, 2015; Pavlenko, 2012), which posits that a decontextualized L2 acquires affective disembodiment and emotional distance. Under this light, self-positivity and other-positivity are automatically processed and embodied to a similar degree in the L1. In the following sections, we examine the mechanisms underlying self-positivity biases.

### 4.1 The controlled and disembodied processing in the L2 context

We found a self-positivity bias in the L2 behavioral performance. Response times in the lexical decision task reflect the accessibility of lexical representations. Specifically, the results showed that in the L2, self-related primes increased the accessibility of positive words relative to other-related primes. We argue that the self-positivity bias arises because self-related information is processed preferentially and that positive emotions form part of self-concept (Chavez et al., 2017; Herbert et al., 2011). Positive information about oneself generally meets expectations, whereas positive information about others may be less expected and may require additional processing, reflected by, on an electrophysiological level, larger N400 amplitude (Fields and Kuperberg, 2015). In our fMRI findings, the reduced RMFG activity in the L2 self-positivity condition, relative to other-positivity, likely reflects the willingness to empathize with others’ emotions. The RMFG is associated with processing the suppression of one’s own experiences when considering the mental states of others (Lieberman, 2007). Moreover, empathy itself has been shown to involve both affective and cognitive components, with the latter engaging prefrontal regions such as the medial PFC (Shamay-Tsoory, 2011). L2 acquisition is often shaped by the decontextualized nature of classroom learning, with limited integration of sensory modalities and verbal conditioning, leading to relatively disembodied representations (Pavlenko, 2012). As a result, processing other-related positive words in an L2 may require additional integration of affective and lexical information, potentially recruiting brain regions associated with empathy (e.g., the RMFG).

### 4.2 The automatic and embodied processing in the L1 context

In the L1, processing both self- and other-related information is more automatic and efficient compared to the L2, reflecting greater processing fluency in the former. Response times and RMFG involvement did not differ significantly between self- and other-positivity conditions in the L1, but the response times on L1 trials were faster than in the L2. This finding may reflect a ceiling effect, suggesting highly automatized processing in the L1. This pattern is consistent with the L1 advantage effect, whereby affective processing in an L1 is more automatic and perceptually prioritized (Pavlenko, 2012). This higher level of automaticity in the L1 may have pushed responses near a functional ceiling, reducing the sensitivity of response times and RMFG measures to detect subtle differences between self- and other-positivity conditions. Our results support the self-positivity effect in an L2 but not in an L1, which is consistent with what van Hugten and van Witteloostuijn (2018) and Liu et al. (2022) have reported. Previous research has shown that L2 lexical access is typically less automatized and therefore slower than L1 lexical access (Favreau and Segalowitz, 1983; Plat et al., 2018). Reduced emotional involvement and increased reliance on controlled processing in an L2 leaves greater room for behavioral variability, thereby allowing the self-positivity bias to emerge.

### 4.3 The RMFG assumes different roles in the L1 and L2 contexts

The RMFG appears to serve different functional roles in L1 and L2 contexts. From the perspective of embodiment, the semantic comprehension in an L1 spontaneously evokes perception and corresponding motor information. However, in an L2, there may be less sensorimotor information along with weaker connections to real-life experiences (Lu and Yang, 2025). As mentioned above, the RMFG is associated with processing self-related information (Fields et al., 2019; Herbert et al., 2011). The activation of the RMFG for L1 self-positivity was larger than L2 self-positivity, which may suggest processing self-related information in an embodied way when doing so in the L1. The activation of the RMFG in the L2 may reflect the integration of affective and lexical information. Taken together, these findings suggest that the RMFG supports flexible processes that vary as a function of language embodiment.

One limitation of the present study is the relatively small number of trials per condition. Future studies should include a larger stimulus set and more repetitions per condition to improve statistical power. The other limitation concerns the relatively low proportion of nonword trials in the lexical decision task. Only 20% of the trials were nonwords, whereas a previous study had used a more balanced proportion of words and nonwords (e.g., Luo et al., 2013). Compared with this study, the lower proportion of nonwords in the present design may have contributed to a ceiling effect, particularly in the L1 condition. Future studies should adopt a more balanced word/nonword ratio to reduce potential response bias.

## 5 Conclusion

The present study is the first to explore the influence of language context on positive biases of self and others using behavioral and fMRI methods. The results revealed a dissociation pattern between L1 and L2: A self-positivity bias was observed in L2, whereas this bias was not significant in L1. This suggests distinct mechanisms across languages that underpin the self-positivity bias: self-positivity bias is processed more embodied in an L1, drawing on richer emotional associations. Whereas it is processed in a disembodied way in an L2, given the need to integrate affective and lexical information. In all, the findings from this study inform our understanding of the cognitive architectures that support emotional influences on a self-positivity bias in L1s and L2s.

## Data Availability

The datasets presented in this study can be found in online repositories. The names of the repository/repositories and accession number(s) can be found at: Kong et al. (2020).
