# Supplementary material for: Neural correlates of affective self-positivity bias in first and second languages
Source: Front Psychol. 2026 Aug 5;17:1749078. doi: 10.3389/fpsyg.2026.1749078 (PMC13485571; doi:10.3389/fpsyg.2026.1749078)
Supplement: Supplementary file 1 [file Supplementary_file_1.docx]

**Supplementary Materials**

**Table S1**

*Real Words and Their Properties Used in the Experiment*

| Positive words | | | | | |
| --- | --- | --- | --- | --- | --- |
| L1 | | | L2 | | |
| Words | Valences | Familiarity | Words | Valences | Familiarity |
| **勇敢** | 7.08 | 7.46 | **brave** | 7.04 | 7.63 |
| **乐观** | 7.25 | 7.92 | **positive** | 7.08 | 7.71 |
| **友好** | 7.38 | 7.50 | **friendly** | 7.13 | 7.92 |
| **仔细** | 5.75 | 7.63 | **careful** | 6.58 | 7.67 |
| **年轻** | 6.29 | 7.50 | **young** | 6.13 | 7.21 |
| **骄傲** | 6.50 | 7.79 | **proud** | 7.54 | 7.42 |
| **有趣** | 6.75 | 7.54 | **funny** | 6.71 | 7.46 |
| **著名** | 5.83 | 6.92 | **famous** | 6.17 | 7.17 |
| **漂亮** | 7.04 | 7.46 | **pretty** | 5.83 | 6.96 |
| Negative words | | | | | |
| L1 | | | L2 | | |
| Words | Valences | Familiarity | Words | Valences | Familiarity |
| **粗心** | 3.71 | 7.50 | **careless** | 3.79 | 7.17 |
| **疯狂** | 4.63 | 6.83 | **crazy** | 4.92 | 7.50 |
| **沮丧** | 3.17 | 7.04 | **frustrated** | 3.58 | 6.88 |
| **孤独** | 2.92 | 7.29 | **lonely** | 3.71 | 6.83 |
| **紧张** | 3.83 | 7.54 | **nervous** | 3.92 | 6.83 |
| **悲伤** | 3.25 | 7.46 | **sad** | 3.58 | 7.58 |
| **愤怒** | 3.25 | 7.63 | **enraged** | 4.00 | 5.25 |
| **饥饿** | 3.75 | 7.54 | **hungry** | 4.00 | 7.29 |
| **胆怯** | 3.79 | 6.50 | **shy** | 4.63 | 7.25 |

**Table S2**

*Nonwords Used in the Experiment*

| **Nonwords** | | | |
| --- | --- | --- | --- |
| **Positive** | | **Negative** | |
| **L1** | **L2** | **L1** | **L2** |
|  | brcve |  | carelass |
|  | friendlg |  | frastrated |
|  | yaung |  | nervaus |
|  | funng |  | emraged |
|  | pratty |  | shg |
|  | pesitive |  | crezy |
|  | caroful |  | lomely |
|  | pioud |  | sud |
|  | fanous |  | hunqry |

**1 Data Analysis of Negative Condition**

1.1 **Behavioral Data Analyses**

For response times (RTs), we conducted a two-way repeated-measures ANOVA with Language (L1, L2) × Identity (Self, Other) as factors in the negative condition. Incorrect responses and RTs that were ± 3 SD from the overall mean were excluded from the data analyses.

1.2 **fMRI Data Analyses**

In a second-level analysis, given that the present study uses a within-subjects design and has different runs assigned to different languages, we used a factorial design (Sui, Rotshtein, & Humphreys, 2013) including four conditions to analyze the interaction between the factors Language and Identity in the negative condition. Statistical maps were thresholded using Gaussian Random Field (GRF) theory. GRF correction belongs to the family-wise error rate corrections approach (Woo et al., 2014) and allows for a strict correction using a single-voxel threshold of p < .005 and a cluster-level threshold of *p* < .05.

2 **Results of Negative Condition**
  2.1 **Behavioral result**

The two-way repeated-measures ANOVA (Language (L1, L2) × Identity (Self, Other) in RT for negative words) revealed a significant main effect of Language, *F*(1,28) = 4.51, *p* = .043, *η_p_^2^* = .139, such that RTs were significantly faster in the L1 (772 ± 29 ms) compared to the L2 (797 ± 24 ms). The main effect of Identity and the interaction of Language × Identity were not significant (*p*s > .05).

2.2 **fMRI result**

The factorial analysis revealed that there were no significant clusters in the two-way interaction between Language (L1, L2) × Identity (Self, Other) after GRF correction (single-voxel threshold of *p* < .005 and a cluster-level threshold of *p* < .05).
